# Neuropsychological test performance of former American football players

**DOI:** 10.1186/s13195-022-01147-9

**Published:** 2023-01-03

**Authors:** Michael L. Alosco, William B. Barr, Sarah J. Banks, Jennifer V. Wethe, Justin B. Miller, Surya Vamsi Pulukuri, Julia Culhane, Yorghos Tripodis, Charles H. Adler, Laura J. Balcer, Charles Bernick, Megan L. Mariani, Robert C. Cantu, David W. Dodick, Michael D. McClean, Rhoda Au, Jesse Mez, Robert W. Turner, Joseph N. Palmisano, Brett Martin, Kaitlin Hartlage, Jeffrey L. Cummings, Eric M. Reiman, Martha E. Shenton, Robert A. Stern, for the DIAGNOSE CTE Research Project, Yi Su, Kewei Chen, Hillary Protas, Connie Boker, Lindsay Farrer, Robert Helm, Douglas I. Katz, Neil Kowall, Gustavo Mercier, James Otis, Jason Weller, Irene Simkin, Alondra Andino, Shannon Conneely, Courtney Diamond, Tessa Fagle, Olivia Haller, Tennyson Hunt, Nicole Gullotti, Brian Mayville, Kathleen McLaughlin, Mary Nanna, Taylor Platt, Fiona Rice, Madison Sestak, Douglas Annis, Christine Chaisson, Diane B. Dixon, Carolyn Finney, Kerrin Gallagher, Jun Lu, Emmanuel Ojo, Brittany Pine, Janani Ramachandran, Sylvain Bouix, Jennifer Fitzsimmons, Alexander P. Lin, Inga K. Koerte, Ofer Pasternak, Hector Arciniega, Tashrif Billah, Elena Bonke, Katherine Breedlove, Eduardo Coello, Michael J. Coleman, Leonhard Jung, Huijun Liao, Maria Loy, Elizabeth Rizzoni, Vivian Schultz, Annelise Silva, Brynn Vessey, Tim L. T. Wiegand, Aaron Ritter, Marwan Sabbagh, Raelynn de la Cruz, Jan Durant, Morgan Golceker, Nicolette Harmon, Kaeson Kaylegian, Rachelle Long, Christin Nance, Priscilla Sandoval, Kenneth L. Marek, Andrew Serrano, Yonas Geda, Bryce Falk, Amy Duffy, Marci Howard, Michelle Montague, Thomas Osgood, Debra Babcock, Patrick Bellgowan, Judith Goldberg, Thomas Wisniewski, Ivan Kirov, Yvonne Lui, Charles Marmar, Lisena Hasanaj, Liliana Serrano, Alhassan Al-Kharafi, Allan George, Sammie Martin, Edward Riley, William Runge, Elaine R. Peskind, Elizabeth Colasurdo, Daniel S. Marcus, Jenny Gurney, Richard Greenwald, Keith A. Johnson

**Affiliations:** 1 Boston University Alzheimer’s Disease Research Center, Boston University CTE Center, Department of Neurology, Boston University Chobanian & Avedisian School of Medicine, Robinson Building, Suite B7800, Boston, MA 02118 USA; 2 Department of Neurology, NYU Grossman School of Medicine, New York, NY USA; 3 Department of Neuroscience, University of California, San Diego, CA USA; 4 Department of Psychiatry, University of California, San Diego, CA USA; 5 Department of Psychiatry and Psychology, Mayo Clinic School of Medicine, Mayo Clinic Arizona, Scottsdale, AZ USA; 6 Cleveland Clinic Lou Ruvo Center for Brain Health, Las Vegas, NV USA; 7 Boston University Alzheimer’s Disease Research Center, Boston University CTE Center, Boston University Chobanian & Avedisian School of Medicine, Boston, MA USA; 8 Department of Biostatistics, Boston University School of Public Health, Boston, MA USA; 9 Department of Neurology, Mayo Clinic College of Medicine, Mayo Clinic Arizona, Scottsdale, AZ USA; 10 Department of Population Health, NYU Grossman School of Medicine, New York, NY USA; 11 Department of Ophthalmology, NYU Grossman School of Medicine, New York, NY USA; 12 Department of Neurology, University of Washington, Seattle, WA USA; 13 Department of Environmental Health, Boston University School of Public Health, Boston, MA USA; 14 Framingham Heart Study, Framingham, MA USA; 15 Slone Epidemiology Center, Boston University, Boston, MA USA; 16 Department of Anatomy & Neurobiology, Boston University Chobanian & Avedisian School of Medicine, Boston, MA USA; 17 Department of Epidemiology, Boston University School of Public Health, Boston, MA USA; 18 Department of Clinical Research & Leadership, The George Washington University School of Medicine & Health Sciences, Washington, DC, USA; 19 Biostatistics and Epidemiology Data Analytics Center (BEDAC), Boston University School of Public Health, Boston, MA USA; 20 Chambers-Grundy Center for Transformative Neuroscience, Department of Brain Health, School of Integrated Health Sciences, University of Nevada Las Vegas, Las Vegas, NV USA; 21 Banner Alzheimer’s Institute, University of Arizona, Arizona State University, Translational Genomics Research Institute, and Arizona Alzheimer’s Consortium, Phoenix, AZ USA; 22 Psychiatry Neuroimaging Laboratory, Department of Psychiatry, Department of Radiology, Brigham and Women’s Hospital, Boston, MA USA; 23 VA Boston Healthcare System, Boston, MA USA; 24 Department of Neurosurgery, Boston University Chobanian & Avedisian School of Medicine, Boston, MA USA

**Keywords:** Alzheimer’s disease, American football, Chronic traumatic encephalopathy, Cognitive function, Neuropsychology, Repetitive head impacts

## Abstract

**Background:**

Patterns of cognitive impairment in former American football players are uncertain because objective neuropsychological data are lacking. This study characterized the neuropsychological test performance of former college and professional football players.

**Methods:**

One hundred seventy male former football players (*n*=111 professional, *n*=59 college; 45–74 years) completed a neuropsychological test battery. Raw scores were converted to *T*-scores using age, sex, and education-adjusted normative data. A *T*-score ≤ 35 defined impairment. A domain was impaired if 2+ scores fell in the impaired range except for the language and visuospatial domains due to the limited number of tests.

**Results:**

Most football players had subjective cognitive concerns. On testing, rates of impairments were greatest for memory (21.2% two tests impaired), especially for recall of unstructured (44.7%) versus structured verbal stimuli (18.8%); 51.8% had one test impaired. 7.1% evidenced impaired executive functions; however, 20.6% had impaired Trail Making Test B. 12.1% evidenced impairments in the attention, visual scanning, and psychomotor speed domain with frequent impairments on Trail Making Test A (18.8%). Other common impairments were on measures of language (i.e., Multilingual Naming Test [21.2%], Animal Fluency [17.1%]) and working memory (Number Span Backward [14.7%]). Impairments on our tasks of visuospatial functions were infrequent.

**Conclusions:**

In this sample of former football players (most of whom had subjective cognitive concerns), there were diffuse impairments on neuropsychological testing with verbal memory being the most frequently impaired domain.

**Supplementary Information:**

The online version contains supplementary material available at 10.1186/s13195-022-01147-9.

## Background

Repetitive head impacts (RHI) from American football have been associated with later-life cognitive symptoms [1–6] and chronic traumatic encephalopathy (CTE) [5, 7–9]. We use the term RHI herein to refer to environmental exposures to repetitive impacts, hits, or blows to the head. These impacts can result in symptomatic traumatic brain injuries (e.g., concussion) and/or less discrete cumulative effects on the brain. The 2021 National Institute of Neurological Disorders and Stroke consensus diagnostic criteria for traumatic encephalopathy syndrome (TES) describe the clinical disorder associated with neuropathologically diagnosed CTE [10]. Impairments in memory and/or executive function are core cognitive features of the TES criteria. The specificity of these impairments to CTE and/or RHI is not clear as similar impairments are known to develop in other neurodegenerative diseases (e.g., Alzheimer’s disease [AD], frontotemporal dementia) [11, 12]. The TES criteria are informed by retrospective reports from informants of brain donors [10, 13] and prospective objective neuropsychological data on individuals exposed to RHI is lacking. The patterns of cognitive impairments in populations at risk for CTE (e.g., former American football players) are not known. This has led to diagnostic challenges for neuropsychologists and other clinicians.

Neuropsychological evaluation is an integral component of the clinical evaluation of neurodegenerative diseases. This is evidenced by clinical diagnostic criteria for neurodegenerative diseases requiring the presence of cognitive impairment on neuropsychological testing [14–18], often defined by standardized test score(s) of 1 to 1.5 standard deviations below the normative mean [14, 17]. The TES research diagnostic criteria emphasize the need for comprehensive neuropsychological testing to substantiate the presence of cognitive impairment [10]. Neuropsychological test scores serve as outcome measures for large-scale multi-center clinical trials of disease-modifying therapies [19]. It is important that cognitive profiles of populations with exposure to RHI, such as former football players, are delineated for research and clinical purposes.

Research studies on the neuropsychological test performance of older former football players have been limited. Schaffert and colleagues conducted a critical review of 22 studies published between 2013 and 2019 on neuropsychological function in former National Football League (NFL) players [3]. Some, but not all, of the studies found evidence for cognitive impairment, most consistently in verbal episodic memory. There were inconsistencies in the domains impaired across studies. That review highlighted the limitations of the research on this topic that include (1) small sample sizes (e.g., *n* = 9 former NFL players); (2) unknown exposure status of the comparison groups; (3) lack of consistent reporting of effect sizes; (4) substantial variation in the extent and quality of the neuropsychological test battery and associated norming practices; and (5) restricted focus on former *professional* American football players.

The objective of this study was to characterize the neuropsychological test performance of a large sample of former college and professional football players from the DIAGNOSE CTE Research Project [20]. Neuropsychological function across major cognitive domains was assessed and we report the sample raw and *T*-scores derived from age, sex, and/or education normative data. Rates of impairment by test and cognitive domain are reported.

## Methods

### Participants and study design

Participants were from the Diagnostics, Imaging, and Genetics Network for the Objective Study and Evaluation of Chronic Traumatic Encephalopathy (DIAGNOSE CTE) Research Project [20]. The objectives of the DIAGNOSE CTE Research Project are to develop in vivo biomarkers for CTE, characterize its clinical presentation, and refine and validate clinical research diagnostic criteria. The study enrolled 240 male participants, ages 45–74, including 120 former NFL players, 60 former college football players, and 60 asymptomatic men without a history of RHI or TBI. All participants volunteered to participate as a part of a research study and they were compensated $500 for their time. Evaluations and participation were not done as part of clinical care or medico-legal purposes. Baseline evaluations were completed between September 2016 and February 2020. Inclusion criteria included no contraindications for MRI, lumbar puncture, or PET procedures; English as the primary language; and consent to all study procedures. Because RHI can often result in TBI, TBI was not exclusionary in the former football players. The former college football players must have played ≥ 6 years of organized football with ≥ 3 years at the college level. Former professional football players must have played ≥ 12 years of organized football, including ≥3 in college and ≥4 seasons in the NFL. Although recruitment for the former football players was *not* based on cognitive (or neuropsychiatric) status, most football players had subjective cognitive concerns at the time of study screening based on the AD8 Dementia Screening Interview and Cognitive Change Index (Supplemental Table 1).

The criteria for the asymptomatic unexposed were no self-reported diagnosed history of TBI of any severity at study screening; no participation in organized contact and collision sports (including American football), military combat, or any other activity that can result in RHI; absence of self-reported formal diagnosis or treatment of psychiatric illness or cognitive impairment; and no self-reported cognitive, behavioral, or mood symptoms at study telephone screening (Supplemental Table 1). They had to have a body mass index ≥24 in order to facilitate matching on body habitus to the former football players. All participants were required to have an informant and adequate decisional capacity at the time of their baseline visit to participate. Additional details of enrollment criteria and recruitment methods have been reported [20]. The neuropsychological test performance of the unexposed asymptomatic men is presented and qualitatively described in the supplemental material (Supplemental Tables 2 and 3). These data are not presented in the main text, and statistical tests that compare the former American football players to the asymptomatic unexposed men on neuropsychological outcomes were not conducted because recruitment of participants for the DIAGNOSE CTE Research Project was based on our risk factor of interest (i.e., elite football play with RHI exposure) and symptoms (i.e., unexposed men must have been asymptomatic at screening). This recruitment strategy was designed for biomarker development [20]. However, it is problematic when examining clinical measures as outcomes because estimates of group differences are magnified.

Participants were evaluated at Boston University Chobanian & Avedisian School of Medicine (with MRI conducted at Brigham and Women’s Hospital); Cleveland Clinic Lou Ruvo Center for Brain Health in Las Vegas, Mayo Clinic Arizona (with PET scans at Banner Alzheimer’s Institute); or NYU Langone Medical Center. Participants underwent a 2-day baseline study visit that included a comprehensive neuropsychological examination and other procedures. All sites received approval by their Institutional Review Board. Participants provided written informed consent. Research was completed in accordance with the Helsinki Declaration.

### Sample size

The final sample size included 59 former college football players and 111 former professional football players. (As shown in the supplement, there were 57 asymptomatic unexposed men.) The sample was reduced after exclusion of participants (across all study groups) for missing data on the primary objective neuropsychological tests (*n*=5) and suboptimal performance validity (*n*=8). Three had missing data on the Golden Stroop Color-Word Test due to colorblindness and were excluded from Golden Stroop Color-Word statistics but were not excluded otherwise.

### Objective neuropsychological evaluation

Participants completed an in-person baseline neuropsychological test battery using standard paper-pencil tests administered by fully trained examiners [20]. A complete list of the domains assessed and neuropsychological tests administered are presented in Table 1.

**Table 1:** DIAGNOSE CTE Research Project Neuropsychological Baseline Measures

Domain	Test/instrument	Normative data source	Demographics used in normative data
Performance validity	Test of Memory Malingering	Tombaugh 1996 [21]	-
Est. premorbid intelligence	Wide Range Achievement Test-Fourth Edition Word Reading subtest	Wilkinson et al. 2006 [22]	Age
Learning and memory	Brief Visuospatial Memory Test–Revised (BVMT-R)	Benedict 1997 [23]	Age
	Neuropsychological Assessment Battery (NAB) List Learning	Stern et al. [24]	Age, sex, Educ.
	Uniform Data Set (UDS) Craft Story 21 Recall	UDS v.3.0, June 2019	Age, sex, Educ.
Executive function	Controlled Oral Word Association Test	Tombaugh et al. 1999 [25]	Age, Educ.
	Golden Stroop Color and Word Interference Test (SCWT)	Golden et al. 2002 [26]	Age, Educ.
	NAB Mazes	Stern et al. [24]	Age, sex, Educ.
	Trail Making Test Part B	UDS v.3.0, June 2019	Age, sex, Educ.
Attention, visual scanning, and psychomotor speed	Symbol Digit Modalities Test	Smith 1973 [27]	Age, Educ
	Trail Making Test Part A	UDS v.3.0, June 2019	Age, sex, Educ.
	UDS Number Span Test	UDS v.3.0, June 2019	Age, sex, Educ.
Language	Animal Fluency	UDS v.3.0, June 2019	Age, Educ.
	UDS Multilingual Naming Test	UDS v.3.0, June 2019	Age, sex, Educ
Visuospatial	Judgment of Line Orientation (odd version)	Woodard et al. [28]	Age
	BVMT-R Copy	Benedict 1997 [23]	-

*Abbreviations*: *UDS* Uniform Data Set, *Educ* education

Neuropsychological measures were selected to assure harmonization with data-sharing platforms, such as the National Alzheimer Coordinating Center (NACC). Many instruments and methodologies that overlap with the NACC Uniform Data Set (UDS) v.3.0 were selected [29, 30]. Measures include those that assess cognitive domains relevant to the features described in neuropathologically confirmed cases of CTE as reported by informants of brain donors [5, 7, 10, 13] and that are part of the TES research diagnostic criteria [10, 13]. Domains assessed included attention, visual scanning, and psychomotor speed; executive functions; learning and episodic memory (verbal and visual); language; and visuospatial abilities. Tests of memory and executive functions were overrepresented given these domains are known to be adversely affected by exposure to RHI [3, 10, 13]. Measures of performance validity and estimated pre-morbid intelligence were administered. Raw scores for all tests were generated according to NACC or test manual protocols. For all tests, the primary raw score outcome was total correct with the exception of Trail Making Test where completion time (in seconds) served as the primary outcome (number of errors is also reported). Neuropsychological test raw scores were converted to *T*-scores using normative data that accounted for age, sex, and education. A small number of tests only accounted for age or age and education. Table 1 provides the normative data source. A *T*-score ≤ 35 (i.e., 1.5 standard deviations [SD] below the normative mean) was considered impaired [14, 17, 31]. The *T*-score range was restricted to 20–80 to limit skewed distributions of the data and outliers.

Suboptimal performance validity was defined by below criterion performance on two out of the following three performance validity measures: Trial 2 of the Test of Memory Malingering (TOMM), reliable number span (modified due to use of the UDS Number Span task), and Neuropsychological Assessment Battery (NAB) List Learning Recognition Hits. Established cutoffs for defining performance validity failure on these measures were used but are not disclosed here to preserve test integrity. While failure of one performance validity test can be indicative of invalidity [32], our decision adheres to the revised Slick criteria (Sherman et al.) for identification of malingered neurocognitive dysfunction that advises failure of 2+ performance validity tests [33]. Of the sample with complete neuropsychological data, 10 (4.2%) had suboptimal performance on the TOMM, 8 (3.4%) had a below cutoff score on the reliable number span, and 50 (21.3%) fell below cutoff on the NAB Recognition Hits trial. Fifty (21.3%) failed at least one performance validity measure, six (2.6%) failed two, and only 2 (0.9%) failed all three. Of note, four participants had one performance validity test missing but were above the cutoff on the other two indices. Taken together, for the current sample, a total of 8 participants were excluded for suboptimal performance validity.

### Sample characteristics

Semi-structured interviews were performed, supplemented by online questionnaires, to collect data on demographics, medical and psychiatric history, athletic history, and other variables not relevant to the present study. An aliquot of whole blood was used for *APOE* genotyping. Race and ethnicity were self-reported. A majority of the sample was Black or White. There was insufficient representation of other racial groups to statistically examine them separately. All racial groups are presented in Table 2.

**Table 2:** Sample characteristics

	Former football players (***n*** = 170)	Former professional (***n*** = 111)	Former college (***n*** = 59)	Asymptomatic unexposed men(***n*** = 57)	***P***-value^**d**^
**Demographics**					
Age, mean (SD) years	57.5 (8.1)	59.6 (7.6)	53.6 (7.7)	59.2 (8.3)	0.17
Education, mean (SD) years	16.7 (1.5)	16.6 (1.1)	17.0 (1.9)	17.4 (3.4)	0.14
Race^a^, *n* (%)					0.58
American Indian or Alaska Native	1.0 (0.6)	1.0 (0.9)	0.0	0.0	
Black or African American	55.0 (32.4)	45.0 (40.5)	10.0 (16.9)	21.0 (36.8)	
Native Hawaiian or other Pacific Islander	0.0	0.0	0.0	1.0 (1.8)	
White	110 (64.7)	63.0 (56.8)	47.0 (79.7)	35 (61.4)	
Multiple races	2.0 (1.2)	1.0 (0.9)	1.0 (1.7)	0.0	
Not reported	2.0 (1.2)	1.0 (0.9)	1.0 (1.7)	0.0	
Ethnicity, *n* (%)					--
Hispanic or Latino	3.0 (1.8)	3.0 (2.7)	0.0	0.0	
**Body mass index**, mean (SD) kg/m^2^	32.5 (4.5)	31.8 (4.3)	33.7 (4.8)	30.8 (4.6)	0.02
**Neurodevelopment**					
Attention-deficit/hyperactivity disorder, *n* (%)	14.0 (8.2)	5.0 (4.5)	9.0 (15.3)	1.0 (1.8)	--
Learning disability, *n* (%)	5.0 (2.9)	2.0 (1.8)	3.0 (5.1)	0.0	--
**Athletic**					
Total years of football, mean (SD) years	15.8 (4.4)	18.0 (3.3)	11.5 (2.6)	-	--
Age of first exposure to football, mean (SD) years	11.1 (2.8)	11.6 (2.7)	10.2 (2.6)	-	--
Primary position at highest level of play, *n* (%)					--
Offensive lineman	42.0 (24.7)	20.0 (18.0)	22.0 (37.3)	-	
Offensive back or receiver	44.0 (25.9)	31.0 (27.9)	13.0 (22.0)	-	
Defensive lineman	19.0 (11.2)	14.0 (12.6)	5.0 (8.5)	-	
Linebacker	27.0 (15.9)	20.0 (18.0)	7.0 (11.9)	-	
Defensive back	34.0 (20.0)	22.0 (19.8)	12.0 (20.3)	-	
Special teams	4.0 (2.4)	4.0 (3.6)	0.0	-	
Reported number of post-definition concussions, median (IQR)^b^	30 (88)	30 (88)	25 (88)	--	--
**APOE genotype**					
ε4 carrier^c^, *n* (%)	49.0 (28.8)	30.0 (27.0)	19.0 (32.2)	11.0 (19.3)	0.21

The sample excluded participants who had suboptimal performance on 2+ performance validity tests and was restricted to participants who had complete data on all primary objective neuropsychological tests (*n* = 13 with missing data [9 former professional, 1 former college, 3 asymptomatic unexposed men])

^a^*n* = 2 with missing data (2 former professional)

^b^All participants were read a definition of concussion prior to being asked to estimate their total number of concussions [34] (*n*=1 with missing data, former professional football player)

^c^*n* = 9 with missing data (4 former professional, 1 former college, 4 asymptomatic unexposed men)

^d^Independent samples *t*-test compared the former American football players and the asymptomatic unexposed men on continuous outcomes and chi-square was used for binary outcomes

### Statistical analyses

Descriptive statistics were used to characterize the neuropsychological test raw and *T*-scores. Rates of impairment on each test and/or test indices are reported, based on above-described cutoffs (e.g., *T*-score ≤ 35). A neuropsychological domain was considered impaired if at least two tests within that domain had a *T*-score ≤ 35. This was only done for the attention, visual scanning, psychomotor speed domain, executive function domain, and the episodic memory domain as there was insufficient number of tests for the language and visuospatial domains (as designed). While we report the frequency of those who had one impaired score, our interpretation of an impaired domain is based on 2+ tests falling below the threshold given the high base rates of test impairments on large batteries among normative individuals [31, 35]. For the memory domain, measures that counted towards impairment included the long delay recall trials from the Brief Visuospatial Memory Test-Revised, Neuropsychological Assessment Battery List Learning task, and Craft Story 21 Recall (Paraphrase). Analysis of covariance controlling for age compared the former college and professional football players on the neuropsychological test raw scores. Statistical analyses were conducted using IBM SPSS Statistics, version 27.

## Results

Sample characteristics are in Table 2. The sample included 170 former American football players (111 former professional football players, 59 former college football players). The sample of football players was 57.5 (SD=8.1) years old and had 16.7 (SD = 1.5) years of education, and 55 (32.4%) were Black or African American. Tables 3 and 4 show the neuropsychological test performance of the former American football players. Analysis of covariance controlling for age showed a statistically significant difference between the former college and professional football player groups on only three of the primary neuropsychological tests, all memory (Supplemental Tables 4 and 5), though the significant differences would not have survived multiple comparison adjustments. For this reason, the former college and professional American football players were combined and described as a single group.

**Table 3:** Baseline neuropsychological test performance of former American football players

Domain	Test/instrument	Former American football players (***n*** = 170)			
		Raw			***T***-score Mean (SD)
		Mean (SD)	Min	Max	
Performance validity	TOMM Trial 2	49.5 (2.4)	32	50	-
	UDS Reliable Number Span	9.9 (2)	6	16	-
	NAB List Learning Recognition	10.4 (1.4)	6	12	37.3%ile (28.2)^a^
Est. premorbid intelligence	WRAT-4^b,c^	62.4 (5.5)	39	70	102.9 (12.7)^b^
Learning and memory	BVMT-R Trials 1–3	20.9 (7.2)	6	35	46.3 (12.6)
	BVMT-R delayed recall	8.6 (2.8)	0	12	49.8 (12.5)
	NAB List Learning trials 1–3	19.6 (4.7)	9	31	40.4 (9.3)
	NAB List Learning SDR	5.7 (2.6)	0	11	39.6 (12.1)
	NAB List Learning LDR	5.2 (2.9)	0	11	38.8 (12.5)
	Craft Story Immediate (Verbatim)	18.8 (6.1)	1	33	44.3 (9.1)
	Craft Story Immediate (Paraphrase)	14.7 (3.9)	1	24	44.8 (9.6)
	Craft Story Delay (Verbatim)	15.7 (6.1)	0	30	43 (9.2)
	Craft Story Delay (Paraphrase)	13.1 (4.2)	0	21	43.2 (9.8)
Executive function	COWAT	41.2 (11.4)	3	76	48 (9.6)
	SCWT Interference^d^	36.6 (9.7)	11	67	48.4 (6.6)
	NAB Mazes	16.0 (6.2)	2	26	53.9 (10.2)
	Trail Making Test Part B	80.4 (43.4)	29	300	44.7 (10.6)
	Trial Making Test Part B errors	0.6 (0.9)	0	4	-
Attention, visual scanning, and psychomotor speed	Symbol Digit Modalities Test	47.4 (9.6)	24	75	48.4 (10.3)
	Trail Making Test Part A	30.2 (10.6)	12	72	44.5 (10.3)
	Trial Making Test Part A errors	0.2 (0.4)	0	2	-
	UDS Number Span Test: Forward	8.5 (2.1)	3	14	49.2 (8.8)
	UDS Number Span Test: FL	6.8 (1.2)	4	9	49.6 (9.6)
	UDS Number Span Test: Backward	6.8 (2.2)	2	13	46.2 (10.1)
	UDS Number Span Test: BL	4.9 (1.3)	2	8	46.2 (10)
Language	Animal Fluency	21 (5.4)	6	35	46 (9.4)
	Multilingual Naming Test	29.2 (2.5)	7	32	43.5 (9.9)
Visuospatial ability	Judgment of Line Orientation (odd version)	12.7 (2.3)	5	15	53 (9.9)
	BVMT-R Copy^a^	11.7 (0.7)	9	12	-

The sample excluded participants who had suboptimal performance on 2+ performance validity tests and was restricted to participants who had complete data on all primary objective neuropsychological tests (across all DIAGNOSE CTE Research Project study groups)

*Abbreviations*: *TOMM* Test of Memory Malingering, *RH* Recognition Hits, *WRAT-4* Wide Range Achievement Test-Fourth Edition Word Reading, *BVMT-R* Brief Visuospatial Memory Test-Revised, *NAB* Neuropsychological Assessment Battery List Learning [*SDR* Short Delay Recall, *LDR* Long Delay Recall], *UDS* Uniform Data Set, *COWAT* Controlled Oral Word Association Test, *SCWT* Golden Stroop Color and Word Test, *FL* Forward Longest Span, *BL* Backward Longest Span

^a^Percentile reported

^b^*n* = 1 former football player with missing data for WRAT-4 and BVMT-R Copy

^c^Standard score reported for WRAT-4

^d^*n* = 2 with missing data due to colorblindness

**Table 4:** *T*-score distributions of baseline neuropsychological test performance of former American football players

Domain	Test/instrument	Former American football players (***n*** = 170), ***n*** (%)			
		≤ 35	36–39	40–49	≥ 50
Learning and memory	BVMT-R Trials 1–3	37 (21.8)	16 (9.4)	44 (25.9)	73 (42.9)
	BVMT-R delayed recall	29 (17.1)	6 (3.5)	41 (24.1)	94 (55.3)
	NAB List Learning trials 1–3	52 (30.6)	19 (11.2)	71 (41.8)	28 (16.5)
	NAB List Learning SDR	64 (37.6)	32 (18.8)	35 (20.6)	39 (22.9)
	NAB List Learning LDR	76 (44.7)	10 (5.9)	47 (27.6)	37 (21.8)
	Craft Story Immediate (Verbatim)	25 (14.7)	25 (14.7)	73 (42.9)	47 (27.6)
	Craft Story Immediate (Paraphrase)	27 (15.9)	19 (11.2)	69 (40.6)	55 (32.4)
	Craft Story Delay (Verbatim)	26 (15.3)	28 (16.5)	78 (45.9)	38 (22.4)
	Craft Story Delay (Paraphrase)	32 (18.8)	16 (9.4)	73 (42.9)	49 (28.8)
Executive function	COWAT	11 (6.5)	23 (13.5)	69 (40.6)	67 (39.4)
	SCWT Interference^a^	6 (3.6)	9 (5.4)	82 (48.8)	71 (42.3)
	NAB Mazes	7 (4.1)	8 (4.7)	41 (24.1)	114 (67.1)
	Trail Making Test Part B	35 (20.6)	12 (7.1)	58 (34.1)	65 (38.2)
Attention, visual scanning, and psychomotor speed	Symbol Digit Modalities Test	20 (11.8)	9 (5.3)	56 (32.9)	85 (50)
	Trail Making Test Part A	32 (18.8)	10 (5.9)	65 (38.2)	63 (37.1)
	UDS Number Span Test: Forward	4 (2.4)	15 (8.8)	74 (43.5)	77 (45.3)
	UDS Number Span Test: FL	14 (8.2)	10 (5.9)	57 (33.5)	89 (52.4)
	UDS Number Span Test: Backward	25 (14.7)	24 (14.1)	62 (36.5)	59 (34.7)
	UDS Number Span Test: BL	24 (14.1)	6 (3.5)	89 (52.4)	51 (30)
Language	Animal Fluency	29 (17.1)	16 (9.4)	71 (41.8)	54 (31.8)
	Multilingual Naming Test	36 (21.2)	18 (10.6)	77 (45.3)	39 (22.9)
Visuospatial	Judgment of Line Orientation (odd version)	12 (7.1)	6 (3.5)	27 (15.9)	125 (73.5)

The sample excluded participants who had suboptimal performance on 2+ performance validity tests and was restricted to participants who had complete data on all primary objective neuropsychological tests

*Abbreviations*: *BVMT-R* Brief Visuospatial Memory Test-Revised, *NAB* Neuropsychological Assessment Battery List Learning [*SDR* Short Delay Recall, *LDR* Long Delay Recall], *UDS* Uniform Data Set, *COWAT* Controlled Oral Word Association Test, *SCWT* Golden Stroop Color and Word Test, *FL* Forward Longest Span, *BL* Backward Longest Span

^a^*n* = 2 with missing data due to colorblindness

As previously described, participants who had suboptimal performance validity on 2+ measures were excluded from the sample (*n*=8). However, there remained four participants who had suboptimal scores on TOMM Trial 2 (scores ranged from 32 to 38). These four participants were retained given their adequate performances on the other two validity tests, including reliable number span (scores ranged from 7 to 11) and NAB List Learning Recognition Hits (percentiles ranged from 13 to 50).

### Estimated premorbid intelligence

Based on the Wide Range Achievement Test, 4th Edition (WRAT-4) standard score, the estimated premorbid intelligence of the former football players fell in the average psychometric range. Fourteen had below average standard scores (i.e., <85). Five (2.9%) reported a diagnostic history of a learning disability, two of whom had a below average standard score on the WRAT-4.

### Learning and episodic memory

The sample mean *T*-scores for NAB List Learning Trials 1–3 and NAB List Learning Short and Long Delay recall trials were all ~40. Thirty-six (21.2%) had impaired episodic memory, representing the domain with the highest rates of impairment. Eighty-eight (51.8%) had at least one test impaired in episodic memory. Of the sample, impairments were frequent on the NAB List Learning Trials 1–3 (30.6%, *n*=52) and on the NAB List Learning Short Delay (37.6%, *n*=64) and Long Delay recall trials (44.7%, *n*=74). The participants recalled a mean of 5.2 (of 12) words after a long delay recall. On the recognition trial, 23 participants (13.8%) had impaired false positive errors (mean = 4.8, SD = 3.8) and 33 participants (19.8%) had impaired recognition hits (mean = 10.4, SD = 1.4).

Compared with learning and memory for unstructured verbal stimuli, learning and memory of structured contextualized information (i.e., a story) were better. The sample mean T-scores were in the average psychometric range for Craft Story 21 Immediate and Delay Recall trials (for both paraphrase and verbatim). Rates of impairments were approximately 15% (*n*=25) for Craft Story 21 Recall Immediate and Delay trials with impairment rates highest for Craft Story 21 Recall Delay Paraphrase (18.8%, *n*=32).

There was better visual than verbal memory test performance. The sample mean *T*-scores on indices of learning and episodic memory for figures (BVMT-R) fell in the average psychometric range. Of the sample, 21.8% (*n*=37) and 17.1% (*n*=29) had impairments on BVMT-R Trials 1–3 and BVMT-R Delay Recall, respectively. Recognition hits (mean = 5.7, SD = 0.7) and false alarms (mean, SD = 0.1, 0.4) were overall intact with few participants having scores in the impaired range (*n* = 6 [3.5%] for hits, *n* = 4 [2.4%] for false alarms).

### Executive functions

Mean *T*-scores were in the average psychometric range and 12 (7.1%) had impaired executive function. Forty-five (26.5%) had a least one test impaired in this domain. Rates of impairments were highest for Trail Making Test Part B (20.6%). On Trail Making Test Part B, 37 had one error, 14 had two errors, and 9 had 2+ errors. Less than 10% of the sample had impaired performance across all other tests.

### Attention, visual scanning, psychomotor speed

The sample mean *T*-scores fell in the average psychometric range for all neuropsychological tests administered in this domain. Twenty-one (12.4%) participants were impaired. Fifty-five (32.4%) had one test impaired and rates of impairments ranged from 2.4% (*n*=4) on UDS Number Span Forward total correct trials to 18.8% (*n*=32) on Trail Making Test Part A. On Trail Making Test Part A, 29 participants had one error and two participants had two errors.

### Language

The sample mean *T*-scores for measures of semantic fluency (Animal Fluency) and confrontation naming (Multilingual Naming Test, MINT) were in the average psychometric range. Regarding rates of impairments, 21.2% (*n*=36) and 17.1% (*n*=29) of the sample were impaired on the MINT and Animal Fluency, respectively.

### Visuospatial

Only 7.1% (*n*=12) were impaired on the Judgment of Line Orientation test. Gross visuospatial abilities on the BVMT-R Copy were intact as raw scores ranged from 9 to 12 (of 12).

### Multidomain impairments

We examined rates of multidomain impairments among the memory, attention, visual scanning and psychomotor speed, and executive function domains. Based on our definition of impairment (i.e., 2+ tests impaired), 25 (14.7%) had 1 domain impaired and 20 (11.8%) of the football players had 2 or more domains that were impaired.

## Discussion

This study examined the neuropsychological test performance of 170 male former college (*n*=59) and professional (*n*=111) football players (ages 45–74), most of whom had subjective cognitive concerns. Impairments were identified using established normative data that account for age, sex, and education. Episodic memory was the most frequently impaired cognitive domain, particularly memory of unstructured verbal information (i.e., NAB List Learning). Compared with unstructured verbal stimuli, learning and recall of contextual verbal stimuli (i.e., Craft stories) and visual information (i.e., BVMT-R figures) were better but impairments still frequent. Other domains with impairments included attention and psychomotor speed (i.e., Trail Making Test Part A) and set-shifting and mental flexibility (Trail Making Test Part B). With the exception of Trail Making Test Part B, performances on tests of executive functions and on visual-perceptual abilities were otherwise preserved.

The results of this study have several implications. Previous research has shown that more than one-third of NFL retirees report being “extremely concerned” about memory and thinking skills [36]. A majority of this sample also had subjective cognitive concerns. Our finding that performance on memory tests was the most frequently impaired is similar to other neuropsychological studies of former NFL players [3]. The mean performance of the word list learning test was at a level of impairment comparable to what is seen in patients with mild cognitive impairment (MCI) [37]. This finding, in combination with less significant reductions on scores on psychomotor speed, confrontation naming, and semantic fluency suggests a neuropsychological profile that resembles an amnestic form of MCI in this sample of former college and professional football players with a mean age of 58, similar to what has been suggested by other investigators [38].

The 2021 NINDS Consensus Diagnostic Criteria for TES include impairments in episodic memory and/or executive functions as core clinical features [10]. One surprising result from this study was that, with the exception of Trail Making Test Part B, performance on tests of executive functions was relatively preserved. While this finding might provide additional support for a neurocognitive profile consistent with amnestic MCI, it might also be an effect of some of the well-known limitations in the neuropsychological assessment of executive functions. Studies that have examined many of the most commonly used tests of executive functions find only modest correlations among the tests suggesting that these functions are difficult to measure as they do not combine neatly into a unitary factor [39]. There are also indications that tests of executive functions often fail to correspond to behavioral ratings of dysexecutive behavior, raising questions about the ecological validity of the measures [40]. In theory, one would expect individuals with the “neurobehavioral dysregulation” of TES to be impaired most specifically on measures of impulsive responding. There was no evidence of impairment in this study on tasks like the Golden Stroop Color Word Interference measure, a well-known index of cognitive impulsivity.

This study included former college and professional football players. There was no statistically significant difference (with consideration of multiple comparisons) between the former college and professional American football players across any of the neuropsychological tests, but there were trends for worse performance in former professional football players. Former professional American football players, and primarily former NFL players, have been the focus of studies on the long-term neuropsychological consequences of American football play [3]. This is one of the first studies to feature middle aged to older adult former *college* football players without subsequent professional experience or other RHI exposure after college. From a public health perspective, it is critical to elucidate the long-term health outcomes of college football players given that approximately 800,000 student athletes have played college football in the USA since 1960, 250,000 of whom are currently older than 60 years of age [41]. Moreover, a recent health outcome survey study found a significantly higher prevalence of cognitive impairment disorders in former college football players compared to the general population, a finding similar to previous studies of former NFL players [41].

A challenge in the field of neuropsychology is the appropriate selection of normative data to derive standardize scores to establish levels of impairment. Here, normative data used included those from the specific test manuals, as well as from the NACC for UDS measures. A majority of normative data accounted for age, sex, and education. However, there were variations in normative adjustments across tests that could have influenced impairment rates by test and domain. Race-based norming was *not* performed. Race-based norming has been incorporated into the training and practice of neuropsychology since at least the 1990s (e.g., Heaton Norms) based on the assumption that race may be a proxy for socioeconomic factors associated with cognitive function. For people who identify as Black, race-based norming results in a stricter threshold needed to be designated as cognitively impaired compared with Whites. The differential treatment of Blacks when scoring and interpreting neuropsychological tests has been a controversial practice [42]. Recently, the NFL ended its use of race-based neuropsychological test norms to determine monetary compensation as part of the NFL Concussion Settlement. The use of race-based norms as part of a rigid algorithm that is void of clinical judgment to determine compensation perpetuates systemic racial injustice and inequity [43]. Prior to consideration of normative data, a majority of neuropsychological tests were developed in White populations, placing Black Americans at initial disadvantage from the beginning. A study is currently underway that is modeling the neuropsychological differences by race in this sample, along with relevant psychosocial, socioeconomic, social, and health factors that might explain observed differences.

There are limitations to the present findings. The asymptomatic unexposed men were required to have no reported symptoms to be eligible for the DIAGNOSE CTE Research Project. While recruitment of the former football players was not based on symptomatic status, most have subjective cognitive (and neuropsychiatric) concerns. This design is appropriate for biomarker development but it limits meaningful comparisons and interpretations on neuropsychological measures between groups as any observed differences could be biased by our recruitment methods. For this reason, statistical comparisons of the former American football players and the asymptomatic unexposed men were not performed [20]. The use of normative data circumvents limitations of study design and informs on rates of neuropsychological impairments among former elite football players. Our ability to make inferences on whether impairments are from pathology and a function of exposure to RHI is challenging given the recruitment design, lack of biomarkers, and in the context of the test performance of the asymptomatic unexposed men. Although impairments were generally infrequent in the unexposed men, approximately 25% and 21% were impaired on BVMT-R Learning Trials and NAB List Learning Long Delay Recall trial, respectively. While the presence of neurological disease in this group cannot be ruled out, it might also be a function of the number of neuropsychological tests administered [31, 44–46]. In the present battery of close to 15 separate correlated indices, rates of impairments in the entire sample might be inflated due to type I error. We also excluded eight participants who had evidence of suboptimal performance on 2+ performance validity tests, based on the revised Slick criteria [33]. Four participants in the sample had suboptimal performance on the TOMM Trial 2 but not on any of the remaining validity indices. We acknowledge that failure of just one validity test can be indicative of invalidity and could have contributed to inflated impairment rates [32]. However, the use of 2+ tests to define invalidity is more stringent, followed recommended guidelines, and performance invalidity rates in this sample were overall low and did not influence the results.

The current study did not include a disease comparison group (e.g., Alzheimer’s disease), which is needed to determine the specificity of the observed neuropsychological profiles and facilitate differential diagnosis. The sample includes individuals who volunteered to participate in research. Most of the male former football players had concerns about their cognitive function, mood, and/or behavior. External validity to the general football population, as well as to women and other athlete populations is limited. We used 1.5 SD below the normative mean to define impairment, a generally accepted convention [14, 17]. We recognize that a continuum exists. Finally, cognitive function was measured using traditional paper-and-pencil tests that might have lacked adequate sensitivity to capture certain impairments. The absence or low rate of impairments in certain domains (e.g., executive functions, visuospatial abilities) might be related to measurement. While digital phenotyping currently lacks clinical applicability, it is an exciting avenue of future research.

## Conclusions

In this sample of 170 male former elite American football players, a comprehensive neuropsychological assessment revealed most frequent impairments in learning and recall for unstructured verbal stimuli. Continued efforts are needed to characterize the neuropsychological profile of individuals exposed to RHI to assist neuropsychologists and other clinicians in disease detection and differential diagnosis. Additional research that includes a disease comparison (e.g., Alzheimer’s disease) and examines causes of neuropsychological impairment in this population is needed. Development of tests sensitive to the specific executive functions disturbed in this population is also an important target for future research. Such development should include and extend beyond traditional paper-and-pencil tests which might not be adequate for the identification of certain impairments in this population.

## Supplementary Information

**Additional file 1: Supplemental Table 1.** Self- and Informant-Reported Symptomatic Status of Participants at Time of Study Screening. **Supplemental Table 2.** Baseline Neuropsychological Test Performance of the Asymptomatic Unexposed Men. **Supplemental Table 3.** T-Score Distributions of Baseline Neuropsychological Test Performance of the Asymptomatic Unexposed Men. **Supplemental Table 4.** Baseline Neuropsychological Test Performance of the Former College and Professional Football Players. **Supplemental Table 5.** T-Score Distributions of Baseline Neuropsychological Test Performance for Former College and Professional Football Players.

## Data Availability

The datasets generated and analyzed during the current study will be available in the Federal Interagency Traumatic Brain Injury Research (FITBIR) repository, https://fitbir.nih.gov. Datasets will also be available through a data*-*sharing portal for the DIAGNOSE CTE Research Project, http://diagnosecte.com. It is also anticipated that study datasets will be available in the Global Alzheimer’s Association Interactive Network (GAAIN) repository, http://www.gaain.org.

## Abbreviations

AD: Alzheimer’s disease
BVMT-R: Brief Visuospatial Memory Test-Revised Version
CTE: Chronic traumatic encephalopathy
FTD: Frontotemporal dementia
MCI: Mild cognitive impairment
NAB: Neuropsychological Assessment Battery
NACC: National Alzheimer’s Coordinating Center
NFL: National Football League
PET: Positron emission tomography
RHI: Repetitive head impacts
SD: Standard deviation
TES: Traumatic encephalopathy syndrome
TOMM: Test of Memory Malingering
UDS: Uniform Data Set
WRAT-4: Wide Range Achievement Test, 4th Edition
