# Supplementary material for: Neuropsychological test performance of former American football players
Source: Alzheimers Res Ther. 2023 Jan 3;15:1. doi: 10.1186/s13195-022-01147-9 (PMC9808953; doi:10.1186/s13195-022-01147-9)
Supplement: Supplementary file 1 — Additional file 1: Supplemental Table 1. Self- and Informant-Reported Symptomatic Status of Participants at Time of Study Screening. Supplemental Table 2. Baseline Neuropsychological Test Performance of the Asymptomatic Unexposed Men. Supplemental Table 3. T-Score Distributions of Baseline Neuropsychological Test Performance of the Asymptomatic Unexposed Men. Supplemental Table 4. Baseline Neuropsychological Test Performance of the Former College and Professional Football Players. Supplemental Table 5. T-Score Distributions of Baseline Neuropsychological Test Performance for Former College and Professional Football Players. [file 13195_2022_1147_MOESM1_ESM.docx]

**Supplemental Material**

**Supplemental Table 1.** Self- and Informant-Reported Symptomatic Status of Participants at Time of Study Screening

**Supplemental Table 2.** Baseline Neuropsychological Test Performance of the Asymptomatic Unexposed Men

**Supplemental Table 3.** T-Score Distributions of Baseline Neuropsychological Test Performance of the Asymptomatic Unexposed Men

**Supplemental Table 4.** Baseline Neuropsychological Test Performance of the Former College and Professional Football Players

**Supplemental Table 5.** T-Score Distributions of Baseline Neuropsychological Test Performance for Former College and Professional Football Players

**Supplemental Table 1.** Self- and Informant-Reported Symptomatic Status of Participants at Time of Study Screening

|  | **AD8-Participant** | **n (%) impaired** | **AD8-Informant** | **n (%) impaired** | **CCI-Participant** | **CCI-Informant** |
| --- | --- | --- | --- | --- | --- | --- |
| **Former Football Players, mean (SD)** | 3.6 (2.9) | 113.0 (66.5) | 3.9 (2.8) | 123.0 (72.4) | 31.6 (12.8) | 31.5 (14.2) |
| **Asymptomatic Unexposed Men, mean (SD)** | 0 (0.2) | 0.0 | 0.1 (0.3) | 0.0 | 13.7 (2.2) | 13.2 (2.1) |

Former football players spanned the symptom continuum and were not recruited based on symptoms. However, the unexposed men were required to not have reported cognitive, behavior, or mood symptoms at time of study screening. Behavior and mood symptoms were screened using internally developed questionnaires and are not presented here given the focus is on cognitive function. The AD8-Dementia Interview was administered by telephone during screening of potential participants and their informants. It is an 8-item checklist of thinking and memory problems and the participant or informant is asked to indicate if there has been a change in the last several years for each item. Scores range from 0-8 and a score of 2 or higher has been shown to predict the presence of mild cognitive impairment. The CCI (Cognitive Change Index) was also administered by telephone during screening of potential participants and their informants. The CCI is a 20-item measure that assesses self and informant perception of cognitive decline. It asks the participants and their informants to rate various aspects of the participants cognitive function compared with the previous five years using a 1 (no change) to 5 (much worse or severe problem) likert scale. Only the first 12 memory questions from the CCI were asked. The range of possible scores is 12 to 60; data on established cutoffs are lacking.

**Supplemental Table 2.** Baseline Neuropsychological Test Performance of the Asymptomatic Unexposed Men

| **Domain** | **Test/Instrument** | **Asymptomatic Unexposed Men**  **(n = 57)** | | | |
| --- | --- | --- | --- | --- | --- |
|  |  | **Raw** | | | **T-score Mean (SD)** |
|  |  | **Mean (SD)** | **Min** | **Max** |  |
| Performance validity | TOMM Trial 2 | 49.9 (0.3) | 48 | 50 | - |
|  | UDS Reliable Number Span | 10.2 (2.5) | 6 | 17 | - |
|  | NAB List Learning Recognition | 10.8 (1.3) | 7 | 12 | 45.6%ile (28.1)^a^ |
| Est. premorbid  intelligence | WRAT-4 | 64.6 (4.1) | 53 | 70 | 109 (11.3)^b^ |
| Learning and memory | BVMT-R trials 1-3 | 20.5 (8) | 3 | 35 | 46.2 (14.2) |
|  | BVMT-R delayed recall | 8.6 (2.8) | 1 | 12 | 50.3 (12.6) |
|  | NAB List Learning trials 1-3 | 21.7 (4.5) | 10 | 31 | 45.8 (9.1) |
|  | NAB List Learning SDR | 6.9 (2.4) | 0 | 11 | 46.6 (11.3) |
|  | NAB List Learning LDR | 6.5 (2.6) | 0 | 11 | 46 (12.4) |
|  | Craft Story Immediate (Verbatim) | 21.1 (6.5) | 7 | 36 | 48 (10.3) |
|  | Craft Story Immediate (Paraphrase) | 15.6 (3.8) | 7 | 22 | 47 (10.1) |
|  | Craft Story Delay (Verbatim) | 18.2 (6.7) | 5 | 31 | 47.2 (10.3) |
|  | Craft Story Delay (Paraphrase) | 14.6 (3.9) | 6 | 22 | 46.9 (9.9) |
| Executive function | COWAT | 41.7 (11) | 15 | 68 | 48.4 (9.6) |
|  | SCWT Interference^c^ | 38.7 (8.1) | 20 | 59 | 48.9 (6.7) |
|  | NAB Mazes | 14.9 (6.5) | 0 | 25 | 53.1 (10.9) |
|  | Trail Making Test Part B | 72.3 (38.2) | 32 | 286 | 47.9 (9.6) |
|  | Trial Making Test Part B errors | 0.4 (1.2) | 0 | 8 | - |
| Attention, visual scanning, & psychomotor speed | Symbol Digit Modalities Test | 46.9 (7.9) | 30 | 67 | 49 (8.4) |
|  | Trail Making Test Part A | 29.0 (9) | 17 | 59 | 46.7 (9.5) |
|  | Trial Making Test Part A errors | 0.1 (0.3) | 0 | 1 | - |
|  | UDS Number Span Test: Forward | 8.5 (2.3) | 5 | 14 | 49.3 (9.7) |
|  | UDS Number Span Test: FL | 6.8 (1.3) | 5 | 9 | 49.2 (10.3) |
|  | UDS Number Span Test: Backward | 7.2 (2.5) | 2 | 14 | 48.7 (10.9) |
|  | UDS Number Span Test: BL | 5.1 (1.3) | 2 | 8 | 47.9 (10) |
| Language | Animal fluency | 21.9 (5.6) | 11 | 36 | 47.8 (10.6) |
|  | Multilingual Naming Test | 30.5 (1.7) | 26 | 32 | 50.2 (10.1) |
| Visuospatial ability | Judgment of Line Orientation (odd version) | 12.5 (2.1) | 8 | 15 | 52.1 (9.3) |
|  | BVMT-R Copy^a^ | 11.6 (0.8) | 9 | 12 | - |

**Note.** The sample excluded participants who had suboptimal performance on 2+ performance validity tests and was restricted to participants who had complete data on all primary objective neuropsychological tests. ^a^Percentile reported; ^b^Standard score reported for WRAT-4; ^c^n = 1 with missing data due to colorblindness

*Abbreviations:* TOMM = Test of Memory Malingering, RH = Recognition Hits, WRAT-4 *=* Wide Range Achievement Test-Fourth Edition Word Reading, BVMT-R = Brief Visuospatial Memory Test-Revised, NAB = Neuropsychological Assessment Battery List Learning [*SDR* Short Delay Recall, *LDR* Long Delay Recall], UDS = Uniform Data Set, COWAT= Controlled Oral Word Association Test, SCWT = Golden Stroop Color and Word Test, FL = Forward Longest Span*,* BL = Backward Longest Span

**Supplemental Table 3.** T-Score Distributions of Baseline Neuropsychological Test Performance of the Asymptomatic Unexposed Men

| **Domain** | **Test/Instrument** | **Asymptomatic Unexposed Men**  **(n = 57), n (%)** | | | |
| --- | --- | --- | --- | --- | --- |
|  |  | **≤ 35** | **36 - 39** | **40 - 49** | **≥ 50** |
| Learning  and memory | BVMT-R trials 1-3 | 14 (24.6) | 8 (14) | 12 (21.1) | 23 (40.4) |
|  | BVMT-R delayed recall | 6 (10.5) | 3 (5.3) | 17 (29.8) | 31 (54.4) |
|  | NAB List Learning trials 1-3 | 8 (14) | 5 (8.8) | 25 (43.9) | 19 (33.3) |
|  | NAB List Learning SDR | 8 (14) | 5 (8.8) | 18 (31.6) | 26 (45.6) |
|  | NAB List Learning LDR | 12 (21.1) | 9 (15.8) | 12 (21.1) | 24 (42.1) |
|  | Craft Story Immediate (Verbatim) | 7 (12.3) | 5 (8.8) | 22 (38.6) | 23 (40.4) |
|  | Craft Story Immediate (Paraphrase) | 6 (10.5) | 7 (12.3) | 21 (36.8) | 23 (40.4) |
|  | Craft Story Delay (Verbatim) | 8 (14) | 6 (10.5) | 21 (36.8) | 22 (38.6) |
|  | Craft Story Delay (Paraphrase) | 8 (14) | 3 (5.3) | 21 (36.8) | 25 (43.9) |
| Executive  function | COWAT | 4 (7) | 4 (7) | 27 (47.4) | 22 (38.6) |
|  | SCWT Interference^a^ | 1 (1.8) | 4 (7.1) | 25 (44.6) | 26 (46.4) |
|  | NAB Mazes | 3 (5.3) | 3 (5.3) | 9 (15.8) | 42 (73.7) |
|  | Trail Making Test Part B | 4 (7) | 5 (8.8) | 20 (35.1) | 28 (49.1) |
| Attention, visual scanning, & psychomotor speed | Symbol Digit Modalities Test | 4 (7) | 5 (8.8) | 16 (28.1) | 32 (56.1) |
|  | Trail Making Test Part A | 8 (14) | 2 (3.5) | 22 (38.6) | 25 (43.9) |
|  | UDS Number Span Test: Forward | 4 (7) | 5 (8.8) | 21 (36.8) | 27 (47.4) |
|  | UDS Number Span Test: FL | 4 (7) | 6 (10.5) | 23 (40.4) | 24 (42.1) |
|  | UDS Number Span Test: Backward | 6 (10.5) | 6 (10.5) | 23 (40.4) | 22 (38.6) |
|  | UDS Number Span Test: BL | 5 (8.8) | 4 (7) | 27 (47.4) | 21 (36.8) |
| Language | Animal fluency | 9 (15.8) | 0 (0) | 24 (42.1) | 24 (42.1) |
|  | Multilingual Naming Test | 7 (12.3) | 1 (1.8) | 15 (26.3) | 34 (59.6) |
| Visuospatial | Judgment of Line Orientation (odd version) | 4 (7) | 4 (7) | 10 (17.5) | 39 (68.4) |

Note. The sample excluded participants who had suboptimal performance on 2+ performance validity tests and was restricted to participants who had complete data on all primary objective neuropsychological tests. ^a^n = 1 with missing data due to colorblindness.

*Abbreviations:* BVMT-R = Brief Visuospatial Memory Test-Revised, NAB = Neuropsychological Assessment Battery List Learning [*SDR* Short Delay Recall, *LDR* Long Delay Recall], UDS = Uniform Data Set, COWAT= Controlled Oral Word Association Test, SCWT = Golden Stroop Color and Word Test, FL = Forward Longest Span*,* BL = Backward Longest Span

**Supplemental Table 4.** Baseline Neuropsychological Test Performance of the Former College and Professional Football Players

| **Domain** | **Test/Instrument** | **Former Professional Football Players**  **(n = 111)** | | | | **Former College Football Players**  **(n = 59)** | | | | **P-value**^d^ |
| --- | --- | --- | --- | --- | --- | --- | --- | --- | --- | --- |
|  |  | **Raw** | | | **T-score Mean (SD)** | **Raw** | | | **T-score Mean (SD)** | -- |
|  |  | **Mean (SD)** | **Min** | **Max** |  | **Mean (SD)** | **Min** | **Max** |  |  |
| Performance validity | TOMM Trial 2 | 49.3 (2.9) | 32 | 50 | - | 49.8 (0.5) | 47 | 50 | - | 0.07 |
|  | UDS Reliable Number Span | 9.8 (2) | 6 | 15 | - | 10.1 (2) | 6 | 16 | - | 0.54 |
|  | NAB List Learning Recognition | 10.3 (1.4) | 6 | 12 | 34.3%ile (28.3)^a^ | 10.6 (1.4) | 7 | 12 | 43%ile (27.5)^a^ | 0.59 |
| Learning and memory | BVMT-R trials 1-3 | 19.6 (7.3) | 6 | 34 | 44.4 (12.9) | 23.4 (6.3) | 7 | 35 | 49.9 (11.2) | 0.09 |
|  | BVMT-R delayed recall | 8.1 (3) | 0 | 12 | 48.2 (13.5) | 9.4 (2.1) | 4 | 12 | 52.8 (9.9) | 0.17 |
|  | NAB List Learning trials 1-3 | 18.6 (4.5) | 9 | 28 | 39.1 (8.9) | 21.3 (4.7) | 9 | 31 | 42.9 (9.4) | 0.02 |
|  | NAB List Learning SDR | 5.3 (2.5) | 0 | 11 | 38.3 (11.4) | 6.4 (2.8) | 0 | 11 | 42.2 (13) | 0.15 |
|  | NAB List Learning LDR | 4.7 (2.7) | 0 | 11 | 37.3 (11.7) | 6.1 (3.1) | 0 | 11 | 41.7 (13.5) | 0.04 |
|  | Craft Story Immediate (Verbatim) | 18 (6) | 1 | 32 | 43.3 (9.1) | 20.4 (6) | 2 | 33 | 46.1 (8.8) | 0.14 |
|  | Craft Story Immediate (Paraphrase) | 14.2 (4) | 1 | 24 | 43.8 (9.9) | 15.6 (3.5) | 3 | 23 | 46.7 (8.7) | 0.17 |
|  | Craft Story Delay (Verbatim) | 14.6 (6) | 0 | 30 | 41.7 (9.3) | 17.7 (5.7) | 0 | 29 | 45.6 (8.5) | 0.04 |
|  | Craft Story Delay (Paraphrase) | 12.5 (4.4) | 0 | 21 | 42 (10.2) | 14.3 (3.5) | 1 | 21 | 45.4 (8.5) | 0.13 |
| Executive function | COWAT | 40.5 (10.9) | 17 | 76 | 47.6 (9.3) | 42.5 (12.2) | 3 | 72 | 48.6 (10.2) | 0.57 |
|  | SCWT Interference^b^ | 36.3 (10.2) | 14 | 67 | 48.2 (7) | 37.3 (8.8) | 11 | 56 | 48.7 (6) | 0.45 |
|  | NAB Mazes | 15.4 (6.5) | 2 | 26 | 54.1 (10.8) | 17.1 (5.3) | 3 | 26 | 53.5 (8.9) | 0.97 |
|  | Trail Making Test Part B | 81.8 (43.1) | 31 | 300 | 44.6 (11) | 77.7 (44.2) | 29 | 300 | 44.8 (10) | 0.74 |
|  | Trial Making Test Part B errors | 0.5 (0.9) | 0 | 4 | - | 0.6 (0.9) | 0 | 4 | - | -- |
| Attention, visual scanning, & psychomotor speed | Symbol Digit Modalities Test | 45.9 (9.8) | 25 | 75 | 47.7 (10.7) | 50.1 (8.5) | 24 | 69 | 49.8 (9.5) | 0.28 |
|  | Trail Making Test Part A | 30.9 (10.7) | 14 | 65 | 44.3 (10.9) | 29 (10.6) | 12 | 72 | 44.9 (9) | 0.50 |
|  | Trial Making Test Part A errors | 0.2 (0.4) | 0 | 1 | - | 0.3 (0.5) | 0 | 2 | - | -- |
|  | UDS Number Span Test: Forward | 8.4 (2.1) | 3 | 14 | 49 (8.8) | 8.7 (2.2) | 4 | 14 | 49.5 (9) | 0.45 |
|  | UDS Number Span Test: FL | 6.8 (1.2) | 4 | 9 | 49.1 (9.3) | 7 (1.3) | 4 | 9 | 50.5 (10) | 0.36 |
|  | UDS Number Span Test: Backward | 6.7 (2.2) | 2 | 12 | 45.7 (10.1) | 7.2 (2.3) | 2 | 13 | 47 (10.1) | 0.23 |
|  | UDS Number Span Test: BL | 4.8 (1.3) | 2 | 8 | 45.5 (10) | 5.2 (1.3) | 2 | 8 | 47.4 (9.9) | 0.14 |
| Language | Animal fluency | 20.3 (4.9) | 11 | 31 | 45.3 (8.8) | 22.4 (6) | 6 | 35 | 47.3 (10.4) | 0.06 |
|  | Multilingual Naming Test | 28.9 (1.8) | 24 | 32 | 41.6 (9.4) | 29.7 (3.4) | 7 | 32 | 47.1 (9.8) | 0.06 |
| Visuospatial ability | Judgment of Line Orientation (odd version) | 12.6 (2.4) | 5 | 15 | 52.4 (10.4) | 12.9 (2.1) | 8 | 15 | 54.1 (9.1) | 0.49 |
|  | BVMT-R Copy^c^ | 11.6 (0.8) | 9 | 12 | - | 11.8 (0.4) | 10 | 12 | - | 0.10 |

The sample excluded participants who had suboptimal performance on 2+ performance validity tests and was restricted to participants who had complete data on all primary objective neuropsychological tests. ^a^Percentile reported; ^b^n = 2 with missing data due to colorblindness (1 former professional, 1 former college); ^c^n = 1 with missing data for BVMT-R Copy (former college); ^d^Analysis of covariance controlling for age compared the former college and professional American football players on raw scores for all tests.

*Abbreviations:* TOMM = Test of Memory Malingering, RH = Recognition Hits, BVMT-R = Brief Visuospatial Memory Test-Revised, NAB = Neuropsychological Assessment Battery List Learning [*SDR* Short Delay Recall, *LDR* Long Delay Recall], UDS = Uniform Data Set, COWAT= Controlled Oral Word Association Test, SCWT = Golden Stroop Color and Word Test, FL = Forward Longest Span*,* BL = Backward Longest Span**Supplemental Table 5.** T-Score Distributions of Baseline Neuropsychological Test Performance for Former College and Professional Football Players

| **Domain** | **Test/Instrument** | **Former Professional Football Players**  **(n = 111), n (%)** | | | | **Former College Football Players**  **(n = 59), n (%)** | | | |
| --- | --- | --- | --- | --- | --- | --- | --- | --- | --- |
|  |  | **≤ 35** | **36 - 39** | **40 - 49** | **≥ 50** | **≤ 35** | **36 - 39** | **40 - 49** | **≥ 50** |
| Learning  and memory | BVMT-R trials 1-3 | 31 (27.9) | 12 (10.8) | 27 (24.3) | 41 (36.9) | 6 (10.2) | 4 (6.8) | 17 (28.8) | 32 (54.2) |
|  | BVMT-R delayed recall | 24 (21.6) | 5 (4.5) | 24 (21.6) | 58 (52.3) | 5 (8.5) | 1 (1.7) | 17 (28.8) | 36 (61) |
|  | NAB List Learning trials 1-3 | 39 (35.1) | 14 (12.6) | 43 (38.7) | 15 (13.5) | 13 (22) | 5 (8.5) | 28 (47.5) | 13 (22) |
|  | NAB List Learning SDR | 45 (40.5) | 24 (21.6) | 21 (18.9) | 21 (18.9) | 19 (32.2) | 8 (13.6) | 14 (23.7) | 18 (30.5) |
|  | NAB List Learning LDR | 56 (50.5) | 8 (7.2) | 30 (27) | 17 (15.3) | 20 (33.9) | 2 (3.4) | 17 (28.8) | 20 (33.9) |
|  | Craft Story Immediate (Verbatim) | 17 (15.3) | 17 (15.3) | 51 (45.9) | 26 (23.4) | 8 (13.6) | 8 (13.6) | 22 (37.3) | 21 (35.6) |
|  | Craft Story Immediate (Paraphrase) | 21 (18.9) | 12 (10.8) | 46 (41.4) | 32 (28.8) | 6 (10.2) | 7 (11.9) | 23 (39) | 23 (39) |
|  | Craft Story Delay (Verbatim) | 21 (18.9) | 21 (18.9) | 48 (43.2) | 21 (18.9) | 5 (8.5) | 7 (11.9) | 30 (50.8) | 17 (28.8) |
|  | Craft Story Delay (Paraphrase) | 25 (22.5) | 12 (10.8) | 47 (42.3) | 27 (24.3) | 7 (11.9) | 4 (6.8) | 26 (44.1) | 22 (37.3) |
| Executive  function | COWAT | 8 (7.2) | 15 (13.5) | 46 (41.4) | 42 (37.8) | 3 (5.1) | 8 (13.6) | 23 (39) | 25 (42.4) |
|  | SCWT Interference^a^ | 5 (4.5) | 5 (4.5) | 57 (51.8) | 43 (39.1) | 1 (1.7) | 4 (6.9) | 25 (43.1) | 28 (48.3) |
|  | NAB Mazes | 5 (4.5) | 7 (6.3) | 23 (20.7) | 76 (68.5) | 2 (3.4) | 1 (1.7) | 18 (30.5) | 38 (64.4) |
|  | Trail Making Test Part B | 25 (22.5) | 8 (7.2) | 32 (28.8) | 46 (41.4) | 10 (16.9) | 4 (6.8) | 26 (44.1) | 19 (32.2) |
| Attention, visual scanning, & psychomotor speed | Symbol Digit Modalities Test | 16 (14.4) | 6 (5.4) | 36 (32.4) | 53 (47.7) | 4 (6.8) | 3 (5.1) | 20 (33.9) | 32 (54.2) |
|  | Trail Making Test Part A | 24 (21.6) | 6 (5.4) | 38 (34.2) | 43 (38.7) | 8 (13.6) | 4 (6.8) | 27 (45.8) | 20 (33.9) |
|  | UDS Number Span Test: Forward | 3 (2.7) | 9 (8.1) | 52 (46.8) | 47 (42.3) | 1 (1.7) | 6 (10.2) | 22 (37.3) | 30 (50.8) |
|  | UDS Number Span Test: FL | 10 (9) | 5 (4.5) | 39 (35.1) | 57 (51.4) | 4 (6.8) | 5 (8.5) | 18 (30.5) | 32 (54.2) |
|  | UDS Number Span Test: Backward | 16 (14.4) | 17 (15.3) | 42 (37.8) | 36 (32.4) | 9 (15.3) | 7 (11.9) | 20 (33.9) | 23 (39) |
|  | UDS Number Span Test: BL | 15 (13.5) | 4 (3.6) | 62 (55.9) | 30 (27) | 9 (15.3) | 2 (3.4) | 27 (45.8) | 21 (35.6) |
| Language | Animal fluency | 18 (16.2) | 12 (10.8) | 52 (46.8) | 29 (26.1) | 11 (18.6) | 4 (6.8) | 19 (32.2) | 25 (42.4) |
|  | Multilingual Naming Test | 30 (27) | 12 (10.8) | 53 (47.7) | 16 (14.4) | 6 (10.2) | 6 (10.2) | 24 (40.7) | 23 (39) |
| Visuospatial | Judgment of Line Orientation (odd version) | 10 (9) | 2 (1.8) | 19 (17.1) | 80 (72.1) | 2 (3.4) | 4 (6.8) | 8 (13.6) | 45 (76.3) |

The sample excluded participants who had suboptimal performance on 2+ performance validity tests and it included participants who had complete data on all primary objective neuropsychological tests. ^a^n = 2 with missing data due to colorblindness (1 former professional, 1 former college).

*Abbreviations:* BVMT-R = Brief Visuospatial Memory Test-Revised, NAB = Neuropsychological Assessment Battery List Learning [*SDR* Short Delay Recall, *LDR* Long Delay Recall], UDS = Uniform Data Set, COWAT= Controlled Oral Word Association Test, SCWT = Golden Stroop Color and Word Test, FL = Forward Longest Span*,* BL = Backward Longest Span
